# Negative effects of a zoanthid competitor limit coral calcification more than ocean acidification

**DOI:** 10.1098/rsos.220760

**Published:** 2022-11-23

**Authors:** Violet E. Doucette, Lucia M. Rodriguez Bravo, Andrew H. Altieri, Maggie D. Johnson

**Affiliations:** ^1^ Department of Biology, Northeastern University, Boston, MA, USA; ^2^ Smithsonian Tropical Research Institute, Apartado 0843-03092, Balboa, Ancon, Republic of Panama; ^3^ Department of Environmental Engineering Sciences, University of Florida, Gainesville, FL, USA; ^4^ Red Sea Research Center, Biological and Environmental Sciences and Engineering Division, King Abdullah University of Science and Technology (KAUST), Thuwal 23955-6900, Kingdom of Saudi Arabia; ^5^ Tenenbaum Marine Observatories Network, Smithsonian Institution, Edgewater, MD, USA

**Keywords:** coral reefs, competition, photophysiology, pH, *Porites*, *Zoanthus*

## Abstract

Ocean acidification (OA) threatens the persistence of reef-building corals and the habitat they provide. While species-specific effects of OA on marine organisms could have cascading effects on ecological interactions like competition, few studies have identified how benthic reef competitors respond to OA. We explored how two common Caribbean competitors, branching *Porites* and a colonial zoanthid (*Zoanthus*), respond to the factorial combination of OA and competition. In the laboratory, we exposed corals, zoanthids and interacting corals and zoanthids to ambient (8.01 ± 0.03) and OA (7.68 ± 0.07) conditions for 60 days. The OA treatment had no measured effect on zoanthids or coral calcification but decreased *Porites* maximum PSII efficiency. Conversely, the competitive interaction significantly decreased *Porites* calcification but had minimal-to-no countereffects on the zoanthid. Although this interaction was not exacerbated by the 60-day OA exposure, environmental changes that enhance zoanthid performance could add to the dominance of zoanthids over corals. The lack of effects of OA on coral calcification indicates that near-term competitive interactions may have more immediate consequences for some corals than future global change scenarios. Disparate consequences of competition have implications for community structure and should be accounted for when evaluating local coral reef trajectories.

## 1. Introduction

Anthropogenic stressors are altering the structure and function of ecosystems worldwide and leading to the degradation of ecologically important marine habitats [1,2]. One of the most significant global environmental changes to date is due to increasing fossil fuel emissions and the accompanying uptake of carbon dioxide (CO_2_) by the ocean, which results in decreasing pH and saturation states of calcium carbonate (CaCO_3_) through the process of ocean acidification (OA) [3]. Ocean acidification may have dire consequences for marine habitats, because it directly influences organismal functions that are tied to physico-chemical properties of seawater (i.e. pH and dissolved CO_2_), such as calcification and primary production [4]. Because of the associated changes in carbonate chemistry, OA generally has disproportionately negative effects on organisms that produce calcareous shells and skeletons relative to non-calcifying taxa [5,6]. Ocean acidification is of particular concern for coral reefs as the physical framework is largely built by calcifying scleractinian corals [7]. Net carbonate dissolution and lower calcification rates under OA contribute to the decline and simplification of coral reef habitat [8].

Although there is some species-specific variation in tolerances to changes in carbonate chemistry associated with OA, coral calcification generally decreases under OA because lower calcium carbonate saturation states make it more difficult for calcifying marine taxa to secrete carbonate shells and skeletons [9,10]. Conversely, the increase in dissolved CO_2_ with OA can stimulate photosynthesis [11]. Because CO_2_ is the primary substrate for photosynthesis, elevated CO_2_ can enhance photosynthetic rates and make more energy available for growth in non-calcifying, photosynthetic marine organisms, without the negative effects seen in calcifying taxa [9,12]. An example of benthic taxa that may benefit from added CO_2_, without the negative effects on calcification, are zoanthids. These zooxanthellate anthozoans are closely related to scleractinian corals but lack a carbonate skeleton. Because they possess symbiotic microalgae they are also photosynthetic and thus provide a model to explore the potential for differential responses to OA due to the presence or absence of calcification and photosynthesis. Little is known about effects of OA on zoanthids. We seek to fill this knowledge gap by evaluating if zoanthids, with no calcification but with photosynthetic symbionts, may respond differently to simulated OA than calcifying corals.

Differential responses to environmental stressors could shift the outcome of competitive interactions if one competitor gains a benefit from the environmental change while the other is either unaffected or negatively affected [13]. However, variability in inter- and intraspecific responses to changes in carbonate chemistry makes it difficult to predict the outcome of ecological interactions under OA without empirical evidence [6,14]. Despite the broader repercussions for community trajectories, relatively few studies have directly examined the relationship between OA and species interactions on the performance of benthic taxa [9]. From those studies, OA appears to amplify the negative outcome of coral interactions with algae, and can worsen or have no effect on the outcome of interactions between corals and sponges [15–17]. However, impacts of OA on coral–zoanthid interactions are largely unknown. Exploring how zoanthids and coral competitors may fare under OA, together and alone, will shed light on how competitive dynamics may influence the manifestation of global change at local scales.

**Figure 1.**
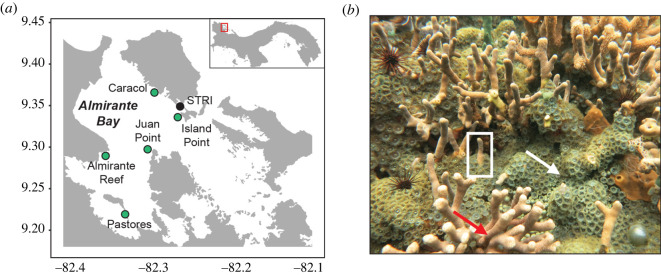
(*a*) Benthic surveys were conducted on coral reefs in Bocas del Toro on the Caribbean coast of Panama (green circles) and the laboratory experiment was conducted at the Smithsonian Tropical Research Institute's (STRI) Bocas del Toro Research Station. (*b*) The branching coral *Porites* (red arrow) and the zoanthid *Zoanthus* (white arrow) are common benthic competitors (white box) on coral reefs in Almirante Bay.

Zoanthids are often abundant and competitively dominant in many benthic subtropical habitats and tropical coral reefs [18,19]. *Zoanthus* sp. is a mat-forming, colonial zoanthid that is common globally and in our Caribbean study system (figure 1). Indeed, these zoanthids are so abundant on reefs in Florida and the greater Caribbean that some shallow intertidal areas are referred to as the ‘*Zoanthus* zone’ [20]. Although competitive dynamics of zoanthids on coral reefs remain poorly studied, their abundance and aggressive competitive strategies suggest they may play a key role in shaping community assemblages in habitats where they are present [18–20].

To quantify the effects of simulated OA on two common benthic reef competitors, we conducted a laboratory experiment in Caribbean Panama with the reef-building branching coral *Porites* sp. and the non-calcifying, photosynthetic zoanthid *Zoanthus* sp. (figure 1*b*). We complemented the laboratory study with field surveys to document the percent cover of each taxon at focal sites in Almirante Bay. The goals of our study were to, 1) document the percent cover of study taxa at representative coral reef sites, 2) determine how calcification and maximum PSII (photosystem II) efficiency of each taxon are influenced by OA and competition and 3) evaluate the potential for OA to exacerbate the competitive interaction between *Porites* sp. and *Zoanthus* sp. By considering how OA effects these reef taxa, alone and when in competition with each other, we can postulate on how a global stressor may influence local-scale community responses in the coming decades.

## 2. Material and methods

### 2.1. Study taxa

Two common benthic taxa on the shallow coral reefs of the Caribbean are the branching coral *Porites* sp. and the mat-forming zoanthid *Zoanthus* sp. (figure 1*b*) [21,22]. Because accurate species identifications of *Porites* are difficult without genetic analyses, we take a conservative approach and refer to all branching species in this genus as *Porites* spp. [23] (hereafter, *Porites*). Similarly, accurate species identifications of zoanthids are also difficult without molecular confirmation [24]. We focused on zoanthids with morphological resemblance to *Zoanthus pulchellus*, and to be conservative we refer to this taxon by the genus name *Zoanthus* spp. (hereafter, *Zoanthus*).

### 2.2. Benthic surveys for percent cover

We conducted this study on the Caribbean coast of Panama in the Bocas del Toro archipelago from February to April 2017. To evaluate the abundance of branching *Porites* and *Zoanthus*, as a proxy for how likely these taxa are to interact, we surveyed five sites around Almirante Bay with benthic photoquadrats (figure 1*a*). These taxa are known to dominate the shallow *Porites* and *Agaricia* dominated reefs in Bocas del Toro [21,22], and to capture this community we focused our surveys at 3–4 m depth. The five shallow coral reef sites were selected because they are part of an established, long-term monitoring program associated with the Smithsonian Tropical Research Institute (STRI) [25] and the Smithsonian's Marine Global Earth Observatory (MarineGEO) monitoring network [26,27].

**Figure 2.**
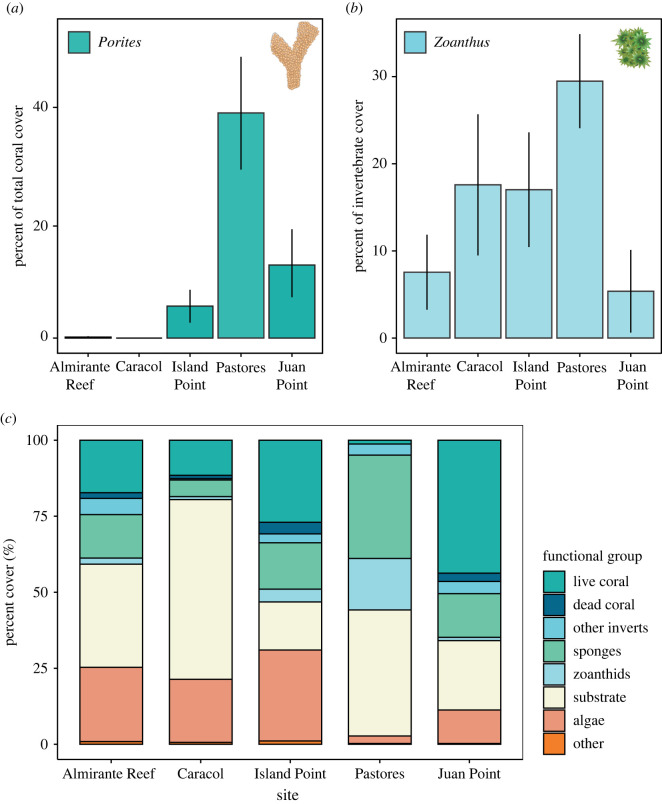
(*a*) Average (± SE) percent of *Porites* relative to all live coral cover and (*b*) of *Zoanthus* relative to all non-coral invertebrates. (*c*) Mean percent cover of the major functional groups at five coral reef sites in Almirante Bay, Bocas del Toro Panama (*N* = 10 or 11 per site).

Photographs were taken of permanent plots (1 m × 0.7 m) positioned every 5 m along a 50 m transect at each of the five sites. Images were analysed by identifying the benthos under 100 randomly stratified points on each photo to the lowest taxonomic resolution possible using CoralNet [28]. Taxa were then categorized into the following groups: live coral, dead coral, sponges, zoanthids, other invertebrates, algae, substrate (e.g. sand and rubble) and other (e.g. transect hardware) (figure 2*c*)*.* Percent cover was averaged across plots within each site to calculate site means per taxa (*N* = 10 or 11 plots per site). To show the relative contribution of the focal taxa to benthic cover by invertebrates on these reefs, we present the abundance of *Porites* as the percent of all coral cover (figure 2*a*) and *Zoanthus* as the percent of all non-coral invertebrate cover (figure 2*b*).

### 2.3. Sample collections for OA experiment

**Figure 3.**
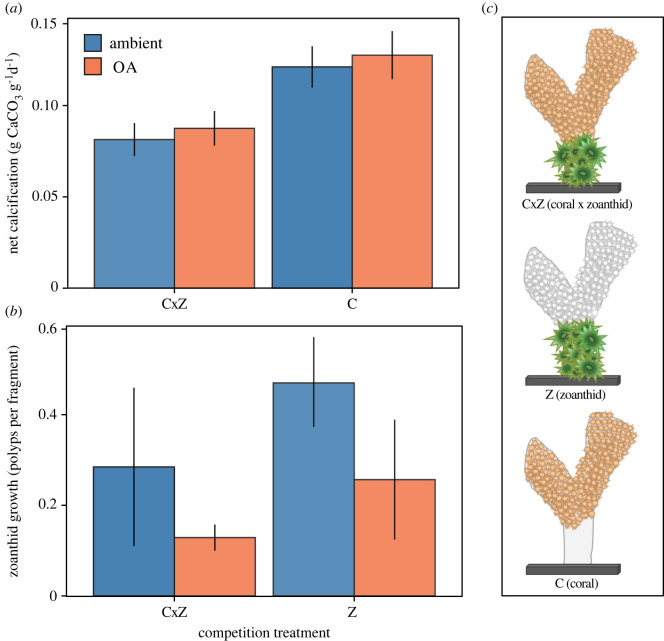
(*a*) Average (± SE) net calcification of *Porites* and (*b*) *Zoanthus* growth by the relative increase in the number of polyps per fragment (change in number of polyps normalized to the initial polyp count). (*c*) Schematic of competition treatments: C is *Porites* alone, Z is *Zoanthus* alone, CxZ is *Porites* and *Zoanthus* together.

Fragments of branching corals (5 cm in length) with a morphology resembling *Porites furcata* were collected with zoanthids (*Zoanthus*) growing at the base (figures 1*b*, 3*c*) from a depth of 2–3 m at Island Point (coordinates: 9.34906, −82.2583). Island Point is one of the long-term monitoring sites and is a reef located <100 m from STRI, which minimized the distance samples were transported after collection (figure 1*a*). We did not differentiate between genotypes in this study and aimed to minimize the potential confounding effects of genotype by selecting fragments from coral colonies that were separated by more than 1 m. Samples were placed in a cooler and immediately transported to wet laboratory facilities where they were maintained in ambient light (282 ± 6 µmol photon m^−2^ s^−1^) and flow-through seawater (29 ± 0.5°C) until the start of the experiment. Prior to the experiment, fragments were cleaned of epiphytes with tweezers and then attached to a plastic base (Vexar) with underwater epoxy (Instant Ocean Holdfast). Fragments were maintained under these conditions for four days to allow for recovery from handling.

### 2.4. Laboratory experimental design

The laboratory experiment was conducted in STRI's Bocas del Toro Research Station (BRS) wet laboratory facilities from February to April 2017. Coral fragments were assigned to one of three interaction treatments for the 60-day experiment: *Porites* with no *Zoanthus* (C), *Zoanthus* with no *Porites* (Z) and *Porites* and *Zoanthus* together (CxZ), (figure 3*c*). For simplicity, this ecological interaction treatment is referred to as the ‘competition’ treatment. The 60-day duration of the experiment is generally an acceptable time frame to determine the initial response of marine taxa to OA in the laboratory [29]. To minimize the potential confounding effects of history of exposure to the competitor across fragments and treatments, we collected coral fragments with an intact coral and zoanthid interaction from the field and then removed the other competitor to create the respective treatments. In the C treatment, *Zoanthus* polyps were removed with tweezers and a soft bristle brush. In the *Z* treatment, coral tissue was carefully removed with a soft bristle brush and air brush. The CxZ fragments remained unaltered with the competitive interaction left intact.

For the OA treatment, we aimed to decrease ambient seawater pH by∼0.3 units, simulating the change in pH expected by the year 2100 in business-as-usual representative concentration pathways (RCPs) for CO_2_ emissions [2]. Although we did not characterize *in situ* pH dynamics at Island Point, the site of coral and zoanthid collection, high-resolution pH data from a nearby reef (Hospital Point, approx. 4.5 km from Island Point) with similar community composition and depth documented the daily average ambient pH at 8.05 ± 0.11 [30]. Therefore, the targeted pH value for the OA treatment in this study was approximately 7.7. Ambient seawater was used for the control treatment and consisted of seawater from the BRS ambient seawater line, which was pumped from a depth of 3 m adjacent to STRI and passed through a 50 µm filter.

The full experiment design consisted of a fully factorial combination of the three competition treatment types (C, Z, CxZ) and the two pH treatments (ambient, OA). The six treatments are referred to as: ambient and coral (Amb-C), ambient and zoanthid (Amb-Z), ambient and coral with zoanthid (Amb-CxZ), OA and coral (OA-C), OA and zoanthid (OA-Z), OA and coral with zoanthid (OA-CxZ). Each of the six treatments were replicated across six tanks, and each tank contained three fragments from one competition treatment (i.e. fragments from different competition treatments were not co-mingled) (*N* = 36 fragments per competition × OA treatment). The replicate fragments within a tank were not independent replicates, and to address issues of pseudoreplication we calculated means across three fragments within a tank and used the means in all analyses (*N* = 6 per competition × OA treatment for each response variable).

### 2.5. Laboratory treatment conditions

The experimental units were 2.8 L plastic tanks that were supplied continuously with either ambient or OA treatment seawater at a rate of approximately 7.0 ml min^−1^. This flow rate yielded approximately 3.6 full seawater exchanges of seawater per day in each tank. Water circulation was further augmented within each tank by mini-aquarium pumps (300 L h^−1^). Tanks were placed within larger shallow water tables that were supplied continuously with ambient seawater and acted as a water bath. Temperature was maintained in the wet laboratory at approximately 28°C, which effectively modulated the temperature of treatment tanks (table 1) and simulated ambient reef temperatures. Although we do not have *in situ* temperature data from the site of collection, ambient temperature from an analogous nearby reef (Hospital Point) during this time of year is 28°C [30].

**Table 1.** Mean physical parameters (± SD) from daily discrete measurements for each pH and competition treatment combination. Temperature, salinity, light, water flow and pH_T_ (total scale pH) were measured every 1–2 days. Replicate tank values (*N* = 6 per treatment) were averaged for each day, and then averaged to yield overall daily treatment means (*N* = 36).

treatment	*T* (°C)	salinity (PSU)	light^a^	flow (ml min^−1^)	pH_T_^b^
Amb-C	27.8 ± 0.54	30.5 ± 0.03	388 ± 156	6.98 ± 1.70	8.01 ± 0.04
Amb-Z	27.7 ± 0.46	30.4 ± 0.13	450 ± 153	6.70 ± 2.61	8.01 ± 0.05
Amb-CxZ	27.7 ± 0.47	30.5 ± 0.04	368 ± 144	6.48 ± 1.78	8.01 ± 0.04
OA-C	27.6 ± 0.54	30.4 ± 0.12	429 ± 172	7.86 ± 2.04	7.74 ± 0.10
OA-Z	27.6 ± 0.48	30.4 ± 0.18	365 ± 125	7.01 ± 1.94	7.74 ± 0.10
OA-CxZ	27.7 ± 0.52	30.5 ± 0.03	392 ± 137	6.41 ± 1.99	7.74 ± 0.11

^a^Light is photosynthetically active radiation (PAR) in µmol photons m^−2^ s^−1^.

^b^pH_T_ is on pH on the total scale.

Lighting was supplied by eight, 7-colour LED aquarium lights (Hydra52, Aquallumination) suspended above experimental tanks. Lights were set to a 12 : 12 H photoperiod (0600–18 : 00), ramping up over four hours starting at dawn and down for four hours prior to dusk to simulate a natural diel cycle. Throughout the experiment, light levels were measured between 1000 and 1400, during the period of maximum light intensity, with a light meter (LiCOR, Li-1400) and 4π spherical quantum sensor placed at the position of coral fragments in tanks. Peak midday irradiance mirrored levels of photosynthetically active radiation (PAR) measured on the day of collection at Island Point (approx. 400 µmol photon m^−2^ s^−1^) with the same meter. To reduce potential unintentional positional effects of tank location within water tables, we haphazardly repositioned the location of each tank underneath the lights every 2–3 days. Epiphyte growth was monitored every 3 days throughout the experiment, and, if discovered, the fragments were cleaned with tweezers and a soft-bristle brush.

The OA treatment was established in three independent reservoir tanks (300 L) that supplied the OA treatment tanks with acidified seawater. The OA manipulation system is described in detail in Johnson *et al*. 2019 [31], and explained briefly here. Reservoirs were continuously provided with ambient seawater and pH was manipulated with pure CO_2_ and maintained at target levels with a pH feedback system. Each reservoir had a laboratory-grade pH probe (Neptune Systems) that measured pH every minute and was connected to an Apex aquacontroller (Neptune Systems) that opened or closed solenoid valves to increase or decrease pH with CO_2_ as necessary to maintain pH within 0.1 units of the target value. Reservoir probes were calibrated weekly with NBS buffers following factory proctol. The target value of reservoirs was cross-calibrated and adjusted based on pH_T_ measured daily. Seawater in reservoirs was mixed continuously with an aquarium pump (1600 l h^−1^) fitted with a venturi injector that facilitated rapid diffusion of CO_2_.

Regular tank measurements were made every 1–2 days between 9 : 30 and 10 : 30 for temperature, pH and salinity, and every 2–3 days for light (table 1). Temperature was measured with a traceable digital thermometer (Thomas Traceable Kangaroo). pH was measured in each tank with a glass triode (Ross Ultra) connected to a pH metre (Orion Star), calibrated daily with certified Tris buffer in synthetic seawater (Batch T30, A. Dickson), and presented as total scale pH (pH_T_). Salinity was measured with a handheld YSI (YSI-63) on water samples collected from reservoir tanks.

We collected discrete water samples every two weeks from reservoir tanks, a subset of treatment tanks, and the ambient seawater line for measurements of total alkalinity (*A*_T_). Samples were either titrated within 12-h of collection or poisoned with 200 µl of a saturated mercuric chloride solution for later processing. *A*_T_ was determined with modified open-cell potentiometric titrations at room temperature using an automated titrator (Mettler Toledo DG115-SC). Titration followed standard operating protocol (SOP) 3b [32], and used certified titrant. The accuracy of *A*_T_ determinations was evaluated by titrating certified reference material (Batch 158, Reference Material for Oceanic CO_2_ measurements, A. Dickson) at the start of titrations, after every 10 titrations and again at the end of each day of titrations. The full carbonate system in seawater was calculated from measured pH_T_, *A*_T_, temperature and salinity with the R package *seacarb* [33], and is presented by treatment in table 2.

**Table 2.** Mean (± s.d.) full carbonate chemistry parameters from bi-weekly discrete bottle samples for the treatments and the OA reservoir. Replicate tanks within a treatment were averaged for each day (*N* = 12 tanks), and these means were averaged to yield overall treatment means (*N* = 4). Total alkalinity (A_T_) was measured every two weeks and pCO_2_ and *Ω*_Ar_ were derived from measured values of temperature, A_T,_ salinity and pH_T_ using the R package *seacarb* [33]. pH_T_ = total scale pH, *Ω* = the saturation state of aragonite, DIC = dissolved inorganic carbon.

treatment	*T* (°C)	salinity (PSU)	pH_T_	*A*_T_ (µmol kg^−1^)	pCO_2_ (µatm)	DIC (µmol kg^−1^)	*Ω*_Ar_
ambient	27.9 ± 0.2	30.8 ± 0.2	8.01 ± 0.03	2252 ± 7	452 ± 37	1986 ± 22	3.24 ± 0.16
OA	27.5 ± 0.6	30.8 ± 0.2	7.68 ± 0.07	2258 ± 8	1086 ± 177	2141 ± 23	1.70 ± 0.18
OA reservoir	28.0 ± 0.2	30.7 ± 0.2	7.68 ± 0.06	2255 ± 12	1100 ± 174	2137 ± 30	1.72 ± 0.22

### 2.6. Laboratory response variables

To determine the net calcification rate, *Porites* fragments were buoyant weighed at the start and end of the experiment [34]. Buoyant weighing effectively quantifies the weight of the calcium carbonate skeleton and does not capture the weight of the living fleshy tissue [34]. Net calcification was determined as the change in weight by converting buoyant weights to dry weights based on the density of calcite (2.71 g cm^−3^) [34]. Calcification rates were normalized to initial fragment weights and are expressed as g per CaCO_3_ per day.

The maximum quantum efficiency of photosystem II (PSII), which we refer to as maximum PSII efficiency (but also known as maximum quantum yield or *F*_v_/*F*_m_), is a non-destructive method of evaluating the performance of coral and zoanthid symbionts [35]. Because these symbiotic relationships break down when the host organism is under stress, this metric can be used as a proxy or indicator of coral or zoanthid ‘health’ [36]. We measured the maximum PSII efficiency of corals and zoanthids with a blue light pulse-amplitude modulated fluorometer (Junior-PAM, Walz) at the end of the experiment. Measurements were taken from fragments that were dark adapted for at least one hour after sunset. For coral measurements, three unique measurements were taken from coral tissue approximately 1 cm from the fragment tip (not in the ‘interaction’ zone on the CxZ fragments). For zoanthids, measurements were taken from three distinct polyps. All measurements were taken with the probe held approximately 0.5 mm away from the tissue surface at a 90° angle. The same PAM settings were used for all measurements (saturation intensity = 12, saturation pulse width = 0.8. measuring light intensity = 8, frequency = 2 and gain = 1) and were selected to optimize initial fluorescence readings (*F*_0_) between 300 and 500.

To evaluate growth of zoanthids, which do not have a carbonate skeleton, we counted the number of zoanthid polyps at the beginning of the experiment and again at the end of the experiment. Polyp counts are expressed as change in the number of polyps per fragment, normalized to the initial number of polyps ((*final – initial*) */ initial*).

After final measurements were collected, coral and zoanthid fragments were frozen for subsequent analyses of symbiont abundance and pigment content. Unfortunately, these analyses were not possible because the fragments thawed following an undetected, major freezer malfunction.

### 2.7. Statistical analyses

Data met assumptions of normality using the Shapiro-Wilk and Levene's tests and all analyses were performed with untransformed data. A two-way fixed factor ANOVA tested the separate and interactive effects of the pH and competition treatments on each response variable. To address issues of pseudoreplication by having multiple fragments from one competition treatment in a tank, we calculated an average for each response value per tank and used this value in subsequent analyses (*N* = 6 per response variable per treatment). Statistical analyses were conducted in R v. 3.4.2 [37]. Raw data and code are archived and available at FigShare [38].

## 3. Results

### 3.1. Benthic cover

*Porites* and *Zoanthus* were present at all five sites surveyed in varying abundances. The overall average (±SE) live coral cover at the sites surveyed in Almirante Bay was 20.2 ± 7.2% (figure 2*c*). *Porites* comprised approximately 19.1 ± 14.2% of all coral cover across the five sites (figure 2*a*) with a range of 0 to 75%, and *Zoanthus* comprised approximately 15.4 ± 4.3% of all non-coral invertebrate cover with a range of 5.4–29.5% (figure 2*b*).

### 3.2. Environmental parameters in experimental tanks

From daily measurements of environmental parameters during the laboratory experiment, the addition of CO_2_ decreased mean ambient seawater pH_T_ from approximately 8.01 in the ambient treatment to approximately 7.74 in the OA treatment (table 1), along with associated changes in carbonate chemistry parameters (table 2). For reference to reported *in situ* values, ambient pH was equivalent to approximately 8.15 and OA pH to approximately 7.88 on the NBS scale.

### 3.3. Net coral calcification

*Porites* calcified significantly less after 60 days of exposure to *Zoanthus* (CxZ treatment), and was unaffected by pH or the interaction of the pH and competition treatments (table 3). The presence of *Zoanthus* decreased net calcification of *Porites* by approximately 27.5% relative to *Porites* alone (C) (figure 3*a*).

**Table 3.** Results of factorial ANOVAs for each response, with pH and competition treatments as fixed factors. Significance at *p* < 0.05 is noted in italics.

treatment	taxa	source	d.f.	*F*	*p*
net calcification	*Porites*	pH	1	0.173	0.681
		competition	1	13.96	*0.001*
		pH × competition	1	0.000	0.990
		residuals	21		
Zoanthid growth (polyp count)	*Zoanthus*	pH	1	1.951	0.177
		competition	1	1.537	0.229
		pH × competition	1	0.051	0.824
		residuals	21		
maximum PSII efficiency	*Porites*	pH	1	163.80	*<0.001*
		competition	1	9.694	*0.005*
		pH × competition	1	4.317	0.050
		residuals	21		
maximum PSII efficiency	*Zoanthus*	pH	1	0.003	0.956
		competition	1	8.877	*0.007*
		pH × competition	1	0.082	0.778
		residuals	21		

### 3.4. Maximum PSII efficiency

**Figure 4.**
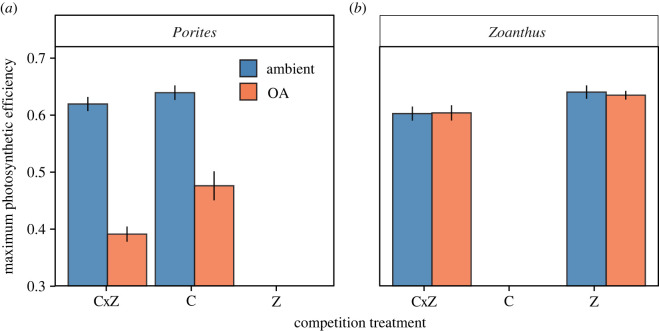
(*a*) Average (± SE) maximum PSII efficiency of *Porites* and (*b*) *Zoanthus* (*N* = 6). Blue represents the ambient treatment and orange the OA treatment. The competition treatments are C for *Porites* alone, Z for *Zoanthus* alone, and CxZ for *Porites* and *Zoanthus* together.

There was a significant negative effect of pH and competition treatments on *Porites* maximum PSII efficiency, and no interactive effect of the two factors (table 3). The most notable response was to pH, where *Porites* PSII efficiency was 32% lower in the OA treatment than in the ambient control (figure 4*a*). In the competition treatment, the presence of *Zoanthus* (CxZ) decreased the PSII efficiency of *Porites* by approximately 10% compared to *Porites* alone.

There were no significant effects of pH or interactive effects of pH and competition treatments on *Zoanthus* (table 3). However, there was a significant negative effect of the competition treatment (table 3), where PSII efficiency of *Zoanthus* decreased by approximately 10% following exposure to *Porites* (CxZ) (table 3, figure 4*b*).

### 3.5. Zoanthid growth

There were no significant effects of the pH or competition treatments on *Zoanthus* growth as estimated by the change in the number of polyps per fragment (figure 3*b*, table 3).

## 4. Discussion

Benthic competition is a fundamental ecological interaction that shapes the structure and function of complex ecosystems, and outcomes of competition can be particularly important in habitats, such as coral reefs, where space is limiting [39]. Our benthic surveys found high abundances of both *Zoanthus* and *Porites* relative to other invertebrate taxa in Almirante Bay, although there was some patchiness within and across sites. The prevalence of the two competitive taxa, combined with *in situ* observations, suggests they are likely in frequent competition with each other. Although zoanthids are known to be dominant competitors [40–42], our results are among the first to demonstrate that the presence of zoanthid competition can significantly decrease coral calcification, which may have implications for the ultimate outcome of that competitive interaction and subsequent community dynamics.

The magnitude of negative zoanthid effects on coral calcification indicates that *Zoanthus* may be a superior competitor on the shallow coral reefs of Bocas del Toro, with the ability to inhibit coral growth under present-day conditions. The decrease in coral calcification may have been due to changes in coral calcification and growth rates, or it could have resulted from enhanced dissolution. For example, *Zoanthus* exposure may have decreased the amount of live coral tissue or elicited an immune response that negatively impacted coral health [43], and these effects could have accumulated over the 60-day experiment and manifested as a decrease in calcification relative to the coral alone treatment. Another possibility is that coral calcification rates may have stayed the same throughout the experiment, but the loss of live coral with zoanthid exposure facilitated skeletal dissolution [44]. However, this seems unlikely because any dissolution would likely have been exacerbated by the lower aragonite saturation state in the OA treatment [45], and there were no measurable effects of OA on coral calcification.

We hypothesized that, due to the lack of carbonate skeleton and presence of photosynthetic symbiont, *Zoanthus* growth would increase under OA while *Porites* growth would be inhibited. However, we found no significant effects of OA treatment on the measured parameters of growth or calcification of either organism in our study. These results are in opposition to the commonly held assumption that calcifying taxa respond negatively to OA, while non-calcifying, photosynthetic taxa respond positively [6,9,46]. Our results contribute to the growing body of literature demonstrating that effects of OA can vary across species, and even within species.

The lack of an OA effect on *Porites* calcification concurs with a suite of studies showing that this genus of coral is generally more resilient than other scleractinians to environmental stressors, including OA [47–51]. Tolerance of *Porites* sp. to OA could be due to biotic or abiotic factors, or a combination of both. For example, history of exposure to pH variability could increase *Porites* tolerance to OA by facilitating adaptations such as the ability to maintain favourable pH levels at the intracellular site of calcification, despite decreasing seawater CaCO_3_ saturation state [52]. Although we detected no effect of OA on changes in buoyant weight, there could have been other skeletal effects that we did not measure (e.g. skeletal extension or density). Growth rates of the non-calcifying zoanthid, as estimated by changes in the number of polyps, were also unaffected by OA. It is possible that zoanthid fleshy biomass per polyp changed, but we were unable to take these measurements due to unexpected loss of samples post-experiment. The lack of OA effects on zoanthid growth is not unexpected given the lack of a carbonate skeleton, though we also found that OA did not enhance zoanthid growth or maximum PSII efficiency. An important caveat to consider with these responses to OA is that the treatments were simulated for 60 days, which may not have been sufficient time to elicit a significant response and does not accurately represent the long-term exposure to decreasing pH that is occurring with OA [53]. As a result, the responses we document here may represent the initial responses of *Porites* and *Zoanthus* to OA and longer time frames should be accounted for in future experiments.

Although there were no effects of OA on calcification, we found negative effects of OA on coral photophysiology. Maximum PSII efficiency in *Porites* decreased under OA, which indicates a decline in coral performance or ‘health’ [37]. Although we observed no visual signs of bleaching, the decrease in maximum PSII efficiency could be indicative of early signs of bleaching [54]. The impacts of OA on coral photophysiology are widely variable throughout the literature, and can range from positive to negative effects [55–57]. The different pattern of response between PSII efficiency and calcification may indicate different temporal scales of *Porites* physiological responses to OA. For example, with longer exposure to acidification, depressed photophysiology or bleaching could eventually lead to reduced coral calcification rates and those physiological effects could compound over time [58]. There was no effect of OA on maximum PSII efficiency in the zoanthid, despite evidence that host-symbiont photosynthetic processes can be altered by exposure to decreasing pH in another species of *Zoanthus* [59,60].

The outcome of competitive interactions has the potential to shift under changing environmental regimes, if new conditions favour one competitor over the other. We found no notable effects of OA on the two competitors, and likewise the effects of competition on *Porites* and *Zoanthus* were not altered by OA. The absence of OA effects on the coral-zoanthid competitive interaction should be interpreted cautiously, as there are myriad other environmental stressors, such as warming, that could influence the outcome of these ecological interactions [60–62]. For example, environmental history and history of exposure to variability in pH can influence organismal responses to subsequent stress exposure [31,63]. Likewise, other environmental changes, such as warming and deoxygenation, are occurring alongside OA and could shift the responses detected in single-stressor experiments [62]. Experiments that incorporate a longer duration of exposure and multiple stressors are the next step for elevating the ecological relevance of laboratory experiments [62].

Although our study was conducted on representative competitors in a Caribbean reef system, our results have broader implications for coral reefs worldwide. Zoanthids are widely distributed on coral reefs where they can be abundant, aggressive competitors of reef-building corals [18,19]. Zoanthids produce toxic secondary compounds (i.e. allelochemicals) and use stinging cells to directly attack tissue of competitors. When combined with rapid asexual growth via clonal budding and fragmentation, these competitive mechanisms may contribute to the dominance of zoanthids over scleractinians [18,42]. Global stressors (i.e. warming and OA) have the potential to indirectly increase competitive efficiency of non-calcifying, photosynthetic, allelopathic competitors like zoanthids simply by enhancing growth while compromising (or not benefiting) their competitors. Such stressors can increase the allelopathic potency of macroalgal competitors over corals [64] and could have similar impacts on zoanthid allelochemicals. Exploring how environmental change influences zoanthid competitive strategies should be studied in further detail to elucidate the specific mechanisms that could further enhance zoanthid growth and competitive abilities over corals. Furthermore, our results on competition between *Porites* and *Zoanthus* were obtained in the laboratory, and additional studies should be conducted to evaluate how responses to competition in the laboratory translate to *in situ* interactions.

Our results provide insight into how zoanthids are coming to dominate the cover of some benthic habitats. Phase shifts toward dominance by soft-bodied cnidarians like zoanthids, along with the loss of corals, are becoming more common globally, particularly in disturbed habitats [65–68]. Enhancement of zoanthid competitive abilities under environmental change, in concert with simultaneous negative effects on reef-building corals, could have long-lasting repercussions for community structure. Although our study did not find disparate effects of OA on *Porites* and *Zoanthus*, this question should continue to be explored in greater detail with other commonly interacting species. Our results have implications for the structure of coral reefs where zoanthid and coral interactions are common and illustrate how increasing abundances of *Zoanthus* could negatively impact the structure and persistence of reef framework builders like *Porites*, with ramifications for ecosystem services like reef accretion and growth. Using a multi-faceted approach in global change experiments that incorporates biotic stressors (i.e. competition) alongside abiotic stressors will allow us to better predict future community trajectories as coral reefs actively respond to environmental change.

## Data Availability

Data are available from Figshare [38].
